# Effect of Electronic Device Addiction on Sleep Quality and Academic Performance Among Health Care Students: Cross-sectional Study

**DOI:** 10.2196/25662

**Published:** 2021-10-06

**Authors:** Sultan Qanash, Faisal Al-Husayni, Haneen Falata, Ohud Halawani, Enas Jahra, Boshra Murshed, Faris Alhejaili, Ala’a Ghabashi, Hashem Alhashmi

**Affiliations:** 1 College of Medicine King Saud bin Abdulaziz University for Health Sciences King Abdulaziz Medical City Jeddah Saudi Arabia; 2 Department of Internal Medicine National Guard Hospital King Abdulaziz Medical City Jeddah Saudi Arabia; 3 College of Medicine King Abdullah International Medical Research Center King Abdulaziz Medical City Jeddah Saudi Arabia; 4 Department of Respiratory Therapy College of Applied Medical Sciences King Saud Bin Abdulaziz University for Health Sciences Jeddah Saudi Arabia; 5 Sleep Medicine and Research Center Faculty of Medicine King Abdulaziz University Jeddah Saudi Arabia

**Keywords:** electronic devices, addiction, sleep quality, grade point average, academic performance, health care students, medical education, sleep, student performance, screen time, well-being

## Abstract

**Background:**

Sleep quality ensures better physical and psychological well-being. It is regulated through endogenous hemostatic, neurogenic, and circadian processes. Nonetheless, environmental and behavioral factors also play a role in sleep hygiene. Electronic device use is increasing rapidly and has been linked to many adverse effects, raising public health concerns.

**Objective:**

This study aimed to investigate the impact of electronic device addiction on sleep quality and academic performance among health care students in Saudi Arabia.

**Methods:**

A descriptive cross-sectional study was conducted from June to December 2019 at 3 universities in Jeddah. Of the 1000 students contacted, 608 students from 5 health sciences disciplines completed the questionnaires. The following outcome measures were used: Smartphone Addiction Scale for Adolescents–short version (SAS-SV), Pittsburgh Sleep Quality Index (PSQI), and grade point average (GPA).

**Results:**

The median age of participants was 21 years, with 71.9% (437/608) being female. Almost all of the cohort used smartphones, and 75.0% (456/608) of them always use them at bedtime. Half of the students (53%) have poor sleep quality, while 32% are addicted to smartphone use. Using multivariable logistic regression, addiction to smartphones (SAS-SV score >31 males and >33 females) was significantly associated with poor sleep quality (PSQI >5) with an odds ratio of 1.8 (1.2-2.7). In addition, male gender and older students (age ≥21 years) were significantly associated with lower GPA (<4.5), with an odds ratio of 1.6 (1.1-2.3) and 2.3 (1.5-3.6), respectively; however, addiction to smartphones and poor sleep quality were not significantly associated with a lower GPA.

**Conclusions:**

Electronic device addiction is associated with increased risk for poor sleep quality; however, electronic device addiction and poor sleep quality are not associated with increased risk for a lower GPA.

## Introduction

Electronic devices such as smartphones, laptops, tablets, personal computers, and televisions have become essential parts of people’s lives due to their easy accessibility and the benefits they offer in facilitating activities of daily living. However, electronic devices can have a detrimental effect on an individual’s health, work, or academic performance if not used in moderation. Serious effects on individual physical and psychological health have been reported with electronic device overuse, such as headache, visual disturbances, chronic neck and back pain, stress, anxiety, and sleep disruption [1-5].

Sleep is a naturally occurring essential process characterized by altered level of consciousness and decreased bodily movement and responsiveness to external stimuli. Sleep is necessary physiologically to restore and maintain physical health and cognitive performance [6-8]. Several factors have been shown to alter sleep and its regulatory processes, including endogenous hemostatic, neurogenic, and circadian processes together with exogenous environmental and behavioral factors [9]. Sleep hygiene and behavioral factors play an essential role in sleep control processes and alter other sleep regulatory mechanisms. Behavioral factors such as drinking coffee, walking around, or talking to someone result in a delay of the sleep process and affect sleep quantity and quality [10]. Poor sleep quality secondary to behavioral factors has been linked to poor or suboptimal academic performance among undergraduate students [11,12].

Electronic devices cause an alteration in sleep architecture, including delayed sleep onset latency and circadian process and decreased rapid eye movement sleep and sleep duration, which are affected by the brightness of screen display in computer and video games when used just before bedtime [13,14]. The bright light exposure from electronic devices at bedtime increases psychophysiological arousals and delays circadian rhythm with other possible neuropsychiatric effects such as depression, anxiety, and night alertness [1-3,15,16].

The negative impact of electronic device use on sleep has been primarily attributed to late-bedtime or longer hours of its use. In 2012, a cross-sectional study found that almost all participants in Norway use electronic devices within 1 hour before sleep [2]. The relationship between mobile phone overuse and disturbances in sleep habits was reported, and the study showed that 61% of adolescents were using mobile phones for 5 hours or more before bedtimes after midnight, and two-thirds of the participants slept less than 6 hours per day [3]. Furthermore, short sleep duration, delayed sleep onset latency, and insomnia were prevalent in adolescents, warranting a public health concern [9]. This link between nocturnal use of electronic devices and sleep insufficiency among adolescents and university students was present in different studies, and the overall use of mobile phones for 5 hours per day was associated with shorter sleep duration and insomnia [1,3,17,18].

Smartphones and other electronic device use have been increasing in recent years among young adults and have become an attribute altering sleep quality and mental health and may affect student academic performance. Therefore, we aimed to study the effect of electronic device addiction on sleep quality and academic performance among health care students at different public universities in Saudi Arabia.

## Methods

### Study Settings

After obtaining approval from the university institutional review boards, the research was conducted at 3 universities in Jeddah: King Saud bin Abdulaziz University for Health Sciences, King Abdulaziz University, and Jeddah University. The study was conducted from June to December 2019. Study participants were the undergraduate health care students at the universities. Students in their internship year were excluded.

### Study Design

This is a cross-sectional study where undergraduate students of health care sciences were invited to participate in completing a self-administrated questionnaire about the effect of electronic devices on their sleep quality and academic performance.

### Sample Size

Assuming that the prevalence of poor sleep is between 40% to 60% and the margin of error between 6% to 10%, the estimated sample size is approximately 600. Sample size calculation was done using PASS 2020 (NCSS LLC) software [19,20].

### Data Collection Methods

The data were collected through an online self-administered questionnaire (Multimedia Appendix 1) comprising 5 major sections. The first section addressed sociodemographic data, including gender, age, and educational discipline. The second section pertained to the type and pattern of electronic device use before bedtime. The grade point average (GPA) used to reflect student academic achievement performance was in the third section. The outcome measures used have good validity and reliability. To measure addiction rate, the Smartphone Addiction Scale for Adolescents–short version (SAS-SV) was used [4]. The scale comprises 5 components: daily life disturbance, withdrawal, cyberspace-oriented relationship, overuse, and tolerance. The cutoff value for males was 31 and females was 33. The values were chosen based on the Haug et al [4] study, with an area under the curve of 0.963 (0.888 to 1.000), sensitivity of 0.875, and specificity of 0.886 for males. For females, the area under the curve was 0.947 (0.887 to 1.000), sensitivity was 0.875, and specificity was 0.886. The Pittsburgh Sleep Quality Index (PSQI) was used to evaluate sleep quality [21]. The PSQI uses 7 areas of measures to differentiate between poor- and good-quality sleepers: subjective sleep quality, sleep latency, sleep duration, habitual sleep efficiency, sleep disturbances, use of sleep medication, and daytime dysfunction over the past month. The PSQI score ranges between 0 to 21, where the higher score represents poorer sleep quality. A total PSQI score of 5 or more indicates poor sleep quality. The invited participants were informed about the study purpose and their voluntary enrollment. Participant information confidentiality was assured.

### Data Analysis

*P*<.05 was considered significant, with a confidence interval of 95%. The collected data were analyzed and managed in SPSS (version 24.0, IBM Corp).

## Results

### Response Rate

An electronic survey was distributed to 1000 students, and 61.80% (608/1000) of participants responded to the questionnaire. There are no missing data regarding the SAS-SV, 8.22% (50/608) of participating students did not enter their GPA, and 16.11% (98/608) of the students had missing data regarding the PSQI. The effects of missing data on the results were checked using multiple imputation, and there was no discrepancy between the results of the analysis of the original data and imputed data. In addition, pooled estimates of the original and imputed data are reported.

### Study Group Characteristics and Electronic Device Use

The median age of the cohort was 21 years with a range from 18 to 40 years. The majority were females (437/608, 71.87%), and the median GPA was 4.5 out of 5. A majority of the students were from the medicine (227/608, 37.3%) and applied medicine (226/608, 37.2%) colleges. One-third (195/608, 32.07%) of the students who completed the SAS-SV were found to be addicted to smartphones, and 62.75% (320/510) of the students who completed the PSQI were considered poor sleepers (Table 1).

**Table 1:** Participant characteristics (n=608).

Variable		Value
**Age (years)**		
	Range	18-40
	Median	21
**Gender, n (%)**		
	Male	171 (28.1)
	Female	437 (71.9)
GPA^a^ (median)		4.5
**Specialty, n (%)**		
	Medicine	227 (37.3)
	Nursing	74 (12.2)
	Dentistry	35 (6.8)
	Pharmacy	46 (7.6)
	Applied medicine	226 (37.2)
**Sleep quality based on PSQI^b^** **, n (%)**		
	Good sleep	190 (37.3)
	Poor sleep	320 (62.7)
**Addiction behavior based on SAS-SV^c^** **, n (%)**		
	Normal	413 (67.9)
	Addicted	195 (32.1)

^a^GPA: grade point average.

^b^PSQI: Pittsburgh Sleep Quality Index.

^c^SAS-SV: Smartphone Addiction Scale for Adolescents–short version.

Habits of electronic device use are demonstrated in Table 2. Almost all (601/608, 98.84%) of the participants use smartphones and half of them use tablets or laptops. Around 19.24% (117/608) of the participants watch TV, and 15.78% (96/608) of the group play video games. Only one participant never used electronic devices before sleep and 67 students rarely did. Three-quarters (456/608, 75.0%) of the cohort always used electronic devices at bedtime, while 18.09% (110/608) reported that they usually did. When asked about putting their devices on silent mode, 44.57% (271/608) of them always did. More than half of the cohort never or rarely wake up due to calls or email at night, and only 5.92% (36/608) said they always wake up. Sleep quality was compared across educational disciplines using a chi-square test. There was no significant difference between disciplines regarding sleep quality (*P*=.50). Poor sleep was similarly prevalent across disciplines ranging between 66.66% (42/63) and 48.27% (14/29; Table 3).

**Table 2:** Electronic device use habits (n=608).

Variable		Value, n (%)
**Type of electronic device**		
	Smartphone	601 (98.8)
	Laptop	333 (54.8)
	Television	117 (19.2)
	Tablet	299 (49.2)
	Video games console	96 (15.8)
**Do you use your device before bed?**		
	Never	1 (0.2)
	Rarely	4 (0.7)
	Sometimes	37 (6.1)
	Usually	110 (18.1)
	Always	456 (75.0)
**Do you put your device on silent before sleep?**		
	Never	99 (16.3)
	Rarely	67 (11.0)
	Sometimes	87 (14.3)
	Usually	84 (13.8)
	Always	271 (44.6)
**Are you woken up by calls or emails at night?**		
	Never	219 (36.0)
	Rarely	170 (28.0)
	Sometimes	128 (21.1)
	Usually	55 (9.0)
	Always	36 (5.9)

**Table 3:** Sleep quality across disciplines (n=190).

Sleep quality	Medicine, n (%)	Applied medicine, n (%)	Pharmacy, n (%)	Nursing, n (%)	Dentistry, n (%)
Good sleep	70 (36.3)	69 (36.7)	15 (40.5)	21 (33.3)	15 (51.7)
Poor sleep	123 (63.7)	119 (63.3)	22 (59.5)	42 (66.7)	14 (48.3)

### Effects on Academic Performance

There was no correlation found between PSQI and GPA (*r*=.018, *P*=.70) or addiction score and GPA (*r*=.02, *P*=.60). Even when GPA, PSQI, and SAS-SV were categorized and included in a logistic regression model along with age and gender to identify GPA predictors, poor sleep and addiction to smartphones were not associated with a lower GPA. However, male gender and age over 21 years were significantly associated (Table 4).

**Table 4:** Predictors of lower grade point average (GPA <4.5).

Predictor	Unadjusted				Adjusted		
	OR^a^	*P* value	95% CI	OR		*P* value	95% CI
Male gender	1.8	.002	1.2-2.5	1.6		.01	1.1-2.3
Age ≥21 years	2.4	.001	1.6-3.8	2.3		.001	1.5-3.7
Poor sleep^b^	1.0	.80	0.7-1.3	0.9		.50	0.6-1.3
Addiction^c^	1.4	.10	0.9-1.9	1.4		.10	0.9-2.0

^a^OR: odds ratio.

^b^Pittsburgh Sleep Quality Index >5.

^c^Smartphone Addiction Scale >33 females and >31 males.

### Effects on Sleep Quality

There is a significant correlation between PSQI and SAS-SV (*r*=.2, *P*<.001). This significant relationship persisted even after GPA, PSQI, and SAS-SV were categorized and included in a logistic regression model along with age and gender to identify predictors of poor sleep. However, GPA, age, and gender were not significantly associated with poor sleep (Table 5).

**Table 5:** Predictors of poor sleep quality (PSQI^a^ >5).

Predictor	Unadjusted				Adjusted		
	OR^b^	*P* value	95% CI	OR		*P* value	95% CI
Male gender	1.0	.80	0.7-1.5	1.0		.90	0.7-1.5
Age ≥21 years	1.2	.40	0.8-1.7	1.2		.40	0.8-1.7
GPA^c^ <4.5	1.0	.80	0.7-1.4	0.9		.50	0.6-1.3
Addiction^d^	1.8	.005	1.2-2.7	1.8		.005	1.2-2.7

^a^PSQI: Pittsburgh Sleep Quality Index.

^b^OR: odds ratio.

^c^GPA: grade point average.

^d^Smartphone Addiction Scale >33 females and >31 males.

## Discussion

### Principal Findings

Our findings suggested that more than half of the study cohort experiences poor sleep quality. The percentage is similar in other studies [22-25]. A recent study by Aldhawyan and colleagues [26] found that 75.4% of the first-year medical students at Imam Abdulrahman bin Faisal University had poor quality of sleep.

Sleep importance cannot be overemphasized due to its critical role in immune, hormonal, and cardiovascular systems in addition to regulating appetite and metabolism [27,28]. The poor sleep quality observed in this study could cause significant health hazards. Sleep disruption mechanisms are believed to have adverse short- and long-term general well-being consequences [29-31]. The activations of the sympathoadrenal system, sympathetic nervous system, and hypothalamic-pituitary-adrenal axis are evident by virtue of multiple observations. Increased oxygen consumption and carbon dioxide production throughout brief and extended arousals during sleep indicate increased metabolism [29].

Moreover, oversecretion of the adrenocorticotropic hormone and altered levels of epinephrine, norepinephrine, and catecholamine are noticed in chronic insomnia [30,31]. Poor sleepers tend to have a higher risk of obesity and type 2 diabetes as suppression of slow-wave sleep leads to decreased insulin sensitivity and leptin levels and increased ghrelin [32,33]. Determining reversible etiologies leading to poor quality of sleep may aid in improving sleep hygiene.

Almost all of the enrolled population uses electronic devices before bedtime to some extent. A significantly positive correlation between the SAS-SV and PSQI was observed. These results support the findings of Van den Bulck et al [34,35] where individuals using electronic devices before bedtime go to bed later and tend to be more tired during the day. Choi et al [36] conducted a study on 2336 high school students on internet overuse and its correlation with excessive daytime sleepiness as a reflection of poor sleep quality. The study found that internet addiction is strongly associated with excessive daytime sleepiness in adolescents, and the prevalence rate of excessive daytime sleepiness for internet addicts was 37.7%, in addition to a higher prevalence of insomnia, witnessed snoring, apnea, teeth grinding, and nightmares. These observations were reported in another study correlating electronic device overuse or addiction with negative consequences on students’ sleep quality and overall health [37].

There are a few suggested mechanisms through which screens affect sleep. Using electronic devices displaces time that could have been spent sleeping. Electronic devices alter bedtime behaviors as users seek more extended screen entertainment, postponing bedtime [38,39]. Furthermore, psychological stimulation from both violent and nonviolent video games increases arousal [40]. Another likely mechanism of negative impact on sleep from electronic device is exposure to the light emitted by screens at bedtime. Melatonin ideally increases in the hours before bedtime, but studies have demonstrated that screen light suppresses its levels in the blood, quelling sleep drive [41]. In addition to melatonin suppression, screen lights prolong the time to fall asleep and reduce the length of rapid eye movement sleep [42].

Minimizing electronic device use, particularly smartphones, around bedtime is expected to ensure better sleep quality. A small randomized pilot study found that sleep quality and working memory improved in participants who restricted mobile phone use for 30 minutes before bedtime [43]. Limiting the use of smartphones before bedtime could improve the quality of sleep. Restricting phone use for just a week increases lights off time by 17 minutes and sleeping time by 21 minutes [44].

Multiple studies have correlated poor academic performance with bad sleeping habits [45-47]. Interestingly, our study has shown that the PSQI score was not associated with a low GPA. However, our findings suggest that poor academic performance is mainly associated with male gender and age of over 21 years. The gender-GPA association has been described before where females had significantly higher grades than males by 6.3% [48]. Similar outcomes were described in another study where females excelled at every academic assessment tool [49]. On the other hand, GPA and age association seems to have different patterns. Sheard [49] concluded that mature‐age students scored higher GPAs compared to young undergraduates, while another study did not show an association between GPA and age [50]. Moreover, older females outperform both older males and younger females in medical school [51].

### Limitations

Missing data related to the PSQI and GPA probably decreased the power of the study to detect a significant association between them. The study was not designed to assess the relationship between sleep quality and outcomes like physical health, psychological well-being, and quality of life. Also, the study lacks an objective way to measure the exact duration of electronic device use. Finally, social desirability bias may be considered with self-reporting of GPA.

### Conclusion

Using electronic devices is common among health care science students and was significantly associated with poor sleep quality. This concern should alert public and academic organizations as more attention is required to motivate students to minimize electronic device use at bedtime to ensure sound sleep hygiene and quality.

## Appendix

Multimedia Appendix 1 Study questionnaire.

## Abbreviations

GPA: grade point average
PSQI: Pittsburgh Sleep Quality Index
SAS-SV: Smartphone Addiction Scale for Adolescents–short version
