# Supplementary material for: Effect of Electronic Device Addiction on Sleep Quality and Academic Performance Among Health Care Students: Cross-sectional Study
Source: JMIR Med Educ. 2021 Oct 6;7(4):e25662. doi: 10.2196/25662 (PMC8529471; doi:10.2196/25662)
Supplement: Multimedia Appendix 1 [file mededu_v7i4e25662_app1.docx]

SECTION 1: Sociodemographic Information

أعلى النموذج

1. What is your gender?

- Female
- Male

2. How old are you?

……………….

3. What is your discipline?

- Medicine
- Applied medical science
- Pharmacy
- Dentistry
- Nursing
- Other (please specify) ………………..

4. What is your GPA?

………………….

SECTION 2: Electronic Devices Use

1. What type of electronic devices do you use? (you can choose more than one)

- Mobile phones
- Laptop
- TV
- Tablet
- Play station

أعلى النموذج

2. Do you put your device beside bed before sleep?

- Always
- Often
- Seldom
- Never

3. Do you use your electronic device before bed?

- Always
- Often
- Seldom
- Never

4. Do you put your device on silent mode before bed?

- Always
- Often
- Seldom
- Never

5. Do you wake up by calls or mails at night?

- Always
- Often
- Seldom
- Never

6. What is the duration of using the device after turning the light off?

- Less than 30 minutes
- 30 mins to 1 hours
- 2 to 3 hours
- More than 3 hours

SECTION 3: The Pittsburgh Sleep Quality Index

During the past month,

1. When have you usually gone to bed?

………….............................................................................................

2. How long (in minutes) has it taken you to fall asleep each night?

………….............................................................................................

3. What time have you usually gotten up in the morning?

………….............................................................................................

4. How many hours of actual sleep did you get at night?

………….............................................................................................

5. How many hours were you in bed?

………….............................................................................................

6. During the past month, how often you had trouble sleeping because you:

أعلى النموذج

A. Cannot get to sleep within 30 minutes

- Not during the past month
- Less than once a week
- Once or twice a week
- Three or more times a week

B. Wake up in the middle of the night or early morning

- Not during the past month
- Less than once a week
- Once or twice a week
- Three or more times a week

C. Have to get up to use the bathroom

- Not during the past month
- Less than once a week
- Once or twice a week
- Three or more times a week

D. Cannot breathe comfortably

- Not during the past month
- Less than once a week
- Once or twice a week
- Three or more times a week

E. Cough or snore loudly

- Not during the past month
- Less than once a week
- Once or twice a week
- Three or more times a week

F. Feel too cold

- Not during the past month
- Less than once a week
- Once or twice a week
- Three or more times a week

G. Feel too hot

- Not during the past month
- Less than once a week
- Once or twice a week
- Three or more times a week

H. Have bad dreams

- Not during the past month
- Less than once a week
- Once or twice a week
- Three or more times a week

I. Have pain

- Not during the past month
- Less than once a week
- Once or twice a week
- Three or more times a week

7. During the past month, how often have you taken medicine (prescribed or “over the counter”) to help you sleep?

- Not during the past month
- Less than once a week
- Once or twice a week
- Three or more times a week

8. During the past month, how often have you had trouble staying awake while driving, eating males, or engaging in social activity?

- Not during the past month
- Less than once a week
- Once or twice a week
- Three or more times a week

9. During the past month, how much of a problem has it been for you to keep up enthusiasm to get things done?

- Not during the past month
- Less than once a week
- Once or twice a week
- Three or more times a week

10. During the past month, how would you rate your sleep quality overall?

- Very good
- Fairly good
- Fairly bad
- Very bad

SECTION 4: Smartphone Addiction Scale

1. Missing planned study due to smartphone use

- Strongly disagree
- Disagree
- Weakly disagree
- Weakly agree
- Agree
- Strongly agree

2. Having a hard time concentrating in class, while doing assignments, or while working due to smartphone use.

- Strongly disagree
- Disagree
- Weakly disagree
- Weakly agree
- Agree
- Strongly agree

3. Feeling pain in the wrists or at the back of the neck while using a smartphone.

- Strongly disagree
- Disagree
- Weakly disagree
- Weakly agree
- Agree
- Strongly agree

4. Won’t be able to stand not having a smartphone.

- Strongly disagree
- Disagree
- Weakly disagree
- Weakly agree
- Agree
- Strongly agree

5. Feeling impatient and fretful when I am not holding my smartphone.

- Strongly disagree
- Disagree
- Weakly disagree
- Weakly agree
- Agree
- Strongly agree

6. Having my smartphone in my mind even when I am not using it.

- Strongly disagree
- Disagree
- Weakly disagree
- Weakly agree
- Agree
- Strongly agree

7. I will never give up using my smartphone even when my daily life is already greatly affected by it.

- Strongly disagree
- Disagree
- Weakly disagree
- Weakly agree
- Agree
- Strongly agree

8. Constantly checking my smartphone so as not to miss conversations between other people on Twitter, Facebook, or whatsApp.

- Strongly disagree
- Disagree
- Weakly disagree
- Weakly agree
- Agree
- Strongly agree

9. Using my smartphone longer than I had intended.

- Strongly disagree
- Disagree
- Weakly disagree
- Weakly agree
- Agree
- Strongly agree

10. The people around me tell me that I use my smartphone too much.

- Strongly disagree
- Disagree
- Weakly disagree
- Weakly agree
- Agree
- Strongly agree
